# Influence of Resin Grade and Mat on Low-Velocity Impact on Composite Applicable in Shipbuilding

**DOI:** 10.3390/polym17030355

**Published:** 2025-01-28

**Authors:** George Cătălin Cristea, Lorena Deleanu, Ioana Gabriela Chiracu, Mihail Boțan, George Ghiocel Ojoc, Alexandru Viorel Vasiliu, Alina Cantaragiu Ceoromila

**Affiliations:** 1 National Institute for Aerospace Research (INCAS) “Elie Carafoli”, 220 Iuliu Maniu, 061126 Bucharest, Romania; botan.mihail@incas.ro; 2 Center of Excellence in Polymer Processing (CE-PP), “Dunarea de Jos” University, 111 Domneasca, 800201 Galati, Romania; ioana.chiracu@ugal.ro (I.G.C.); alexandru.vasiliu@ugal.ro (A.V.V.); alina.cantaragiu@ugal.ro (A.C.C.); 3 Autonomous Flight Technologies, 1 Aeroportului, 077060 Clinceni, Romania; george.ojoc@gmail.com

**Keywords:** low-velocity impact, glass fiber composite, mat, resin, impact energy, maximum force

## Abstract

In this study, the composition and mechanical properties of composites designed for shipbuilding are described. Four different composites were designed and fabricated by the research team, using quadriaxial glass fiber fabric (eight layers in all composites), two different resins (the epoxy resin SikaBiresin^®^ CR82 with the hardener CH80-2 or the polyester resin Enydyne H 68372 TA with Metox-50 W as the accelerator), and a middle layer of Coremat Xi 3 (only applied in some composites). The experimental results of low-velocity impact tests are also discussed, including the graphics force (displacement) and absorbed energy (displacement and velocity). The displacement and composite quality were evaluated through several parameters, such as maximum force, absorbed energy, and maximum displacement. Impact tests were carried out using four impact energy values (50–200 J), with an average impact velocity in the range of 4.37 ± 0.05 m/s. Only partial penetrations were obtained for all tested composites. For the low energy tests (50 J), the four composite materials were not well differentiated by graph shapes and parameter values, but for the higher energy tests, the composites containing Coremat Xi 3 displayed better behavior, having F_max_ reduced with 10.8% to 29.08%. The higher absorbed energy of these composites can be explained by the plateau generated by the force from a longer impactor displacement in contact with the composite. The results generated in this study confirm the suitability of the designed composites for shipbuilding applications. Still, the composites have light differences in terms of energy absorption in low-velocity impact and a significant reduction in maximum force.

## 1. Introduction

Composite materials are widely used in various industries due to their remarkable advantages such as high stiffness and relatively low weight. These innovative materials offer a unique combination of properties that make them the preferred choice in a variety of applications [1,2,3,4]. Their response to dynamic loads is of particular interest, as these materials are used in high-risk systems such as aerospace equipment, marine and off-shore industries [5,6,7], and equipment for intervention in extreme conditions (military applications [8], heavy and automotive industries [9], security in transport and production [10,11,12], etc.). For low-velocity impact applications, such as bumpers for automotive and sports equipment, composites offer additional advantages over traditional materials (i.e., metal alloys or plastics). Composite characteristics can be tailored at different scales, from nano to macro; e.g., nano constituents that can modify the resin and reinforcements, but especially the interface properties, fibers as micro constituents and layers, and fabrics as macro components [13].

Knowledge of the dynamic response of a structure and its resistance to damage is necessary to optimize its use in applications requiring high safety, such as the structural applications of aircraft, ships, or protection systems [14]. For instance, composite crash barriers like car fenders and ship hulls must absorb energy without total fracture. The most effective materials are those that deform plastically, absorbing energy that produces permanent (plastic) deformations, but they are not reusable. In contrast, fenders that remain elastic are able to spring back after impact. For practical reasons, the material must have a modulus greater than 10 GPa. Nonmetallic materials are often used to produce elastic fenders; the overriding consideration is that the displacement before fracture is as great as possible, as the constraint on the modulus ensures that it absorbs enough energy [15].

Composites undergo internal failure due to low-velocity impact loads, leading to a decrease in residual strength and service life. These low-velocity impacts typically occur during maintenance and service activities, but also in the exploitation of systems, with a significant risk of damage. Low-velocity events typically range between 1 m/s (or less) and 10 m/s. Low-velocity impact damage is often undetectable by visual inspection, which compromises the structural stability over time.

The tests reported in this paper focus on low-velocity impacts. As reported in the literature, low velocity is considered (but not restricted) to be about 10 m/s. Our team also conducted tests on similar composites under a ballistic impact, which involved a projectile of 9 mm FMJ at around 400 m/s, considered to be a medium-to-high impact velocity [16]. Other impact tests have been carried out at an impact velocity of 4.32–4.42 m/s, a value that could characterize a collision between ship bodies. For instance, Zhang S. [17] presented a case for a ship collision, with an impact velocity of 4.5 m/s. Zhu Z. et al. [18] reported tests for impacts under 4.98 m/s, and Ni K. et al. [19] conducted similar experimental research for low-velocity impacts at 1–10 m/s and a simulation for 5 m/s. Long B. et al. [20] carried out impact tests at 1.24–3.28 m/s, and Lin H. et al. [21] considered a velocity of 10 m/s, simulating the impact of a ship. Kim S.J. et al. [22] presented scenarios for ship collision at 2.5–5.1 m/s.

Fiber-reinforced polymers are very important for engineering applications due to their high specific stiffness, fatigue performance, good chemical and thermal resistance, and low cost [23]. Due to these advantages, fiber-reinforced composites have been used to replace traditional metallic materials and are widely used in aircraft [24,25], marine structures [26], defense industries [27], automotive structures [28,29], sports equipment [30,31], land transportation [32,33], and construction [34].

Unlike monolithic metallic materials, fibrous composites are used as laminated products with different layered orientations, and each layer is composed of fiber and matrix constituents. Therefore, the failure modes of fibrous composites are more complicated. In order to explore the potential of composite materials in structural design, it is important to analyze and understand their failure mechanisms [35,36,37].

Glass fiber-reinforced epoxy composites have been studied for their particular response to low-velocity impacts [38,39,40]. Yang K. et al. [38] proposed a diagram of impact strength (in kJ/m^2^) as a function of the fraction volume of fibers and fiber type (glass, carbon, silk, etc.). The application field of each fiber composites also depends on the matrix nature. Fiberglass in polyester matrices has lower values for impact strength, and fiberglass in epoxy matrices has the highest values (around 280 kJ/m^2^) for a fiber fraction volume of 60–62%.

Khan et al. conducted impact tests on pipes made of a fiberglass–vinyl ester composite and a fiberglass–epoxy matrix composite, and found that the energy required to initiate damage was higher for the epoxy resin-based pipes compared to the vinyl ester-based pipes. Low velocity impacts can induce significant failure/damage in the composite, causing a severe reduction in the mechanical properties of the composites and a premature and hard-to-predict failure [41].

Reyes G. and Sharma U. studied the low-velocity impact behavior of fiberglass and polypropylene thermoplastic laminates, with excellent energy absorption capabilities in both tested configurations [42]. Mars J. et al. conducted a numerical study on glass fiber-reinforced polyamide composites, finding that the impact velocity had considerable effects on the impact response of composite plates [43]. Venkategowda C. et al. investigated the low-velocity impact response of glass/carbon–epoxy hybrid composites, revealing that the presence of defects significantly affected the energy absorbed by the composites [44].

Structural analysis and the optimization of correct and efficient procedures are very essential to achieving suitable materials [45]. The common optimal design objective of laminated composite structures [46] is to determine the layer thickness, orientations, and number of layers that give the minimum weight of the structure and meet both the imposed constraints and the adopted yield criterion.

Hojo M. et al. [47] made laminated plates of fiberglass and polyester fabric in two thickness variants for low-velocity impact testing up to 1500 J, which is the highest value we found in the literature for tests at low velocity impact. Their results revealed that failure has a delamination component, and the authors concluded that, if the specimens had the same diameters, the impact forces and those determined in quasi-static tests could be scaled according to the thickness of the material.

Kinvi-Dossou G. et al. [48] tested composites made of the same fabric, but with five different matrixes. The samples had dimensions of 2 mm × 100 mm × 150 mm and the striker was 16 mm in diameter. Three tests were carried out for the same conditions (material and energy level), for an impact energy range of 10–60 J. The authors pointed out differences among tested materials for the ratio of maximum force/maximum displacement during impact.

Vescovini A. et al. [49] produced samples made of 8-Harness Satin S2-Glass Fabrics (302 g/m^2^, 0.24 mm, 2.49 g/cm^3^) in an elastomeric polyurethane resin matrix, with 2.48–6.39 mm thickness. The authors tested the impact behavior of these samples with a hemispherical impactor of 16 mm, at different energy impact levels (48 J, 86 J, and 115 J). Only one test was carried out for each set of parameters (material thickness and energy level), but the influence of these two variables on maximum force and displacement was established, and the absorbed energy and the typical failures were described.

AlOmari A.S. et al. [50] studied hybrid composites with glass, carbon, and aramid fabrics under an impact of 20 J (impact velocity of 2.06 m/s) and compared them to simple glass fiber composites in an epoxy matrix, with a thickness of 4.9–7.4 mm. The authors used INSTRON Dynatup 9250 G drop tower, with a hemispherical impactor of 25.4 mm. The glass fiber composites absorbed 9–11.08 J, depending on their thickness, which is lower than carbon composites and even hybrid composites.

Al-Azzawi A.S.M. et al. [51] used S2-glass fiber 32/FM94-epoxy unidirectional prepreg for composites, stacked in [0°/90°/90°/0°] 4s orientations. The produced panels were cured at 120 °C and a pressure of 0.6 MPa. The panels were square, with dimensions of 4.25 mm × 240 mm × 240 mm, and were then cut into 70 mm square plates. An impactor with a 20 mm spherical diameter was used with a CEAST 9350 impact machine. Each test was repeated three times, and conducted at ambient (25 °C), high (50 °C, 75 °C, 100 °C), and low temperatures (−25 °C, −55 °C), at various impact energies (75 J, 150 J, 225 J). The impact load, deflection, and energy absorption were analyzed. The composites were stiffer and exhibited less damage at cryogenic temperatures than they did at other temperatures. The matrix softened at high temperatures, with an enlarged damaged area. Total penetration occurred at higher impact energy levels and at lower temperatures.

A recent research study on a composite made of the same quadriaxial fabric and bicomponent resin SikaBiresin^®^ CR82 with the CH80-2 hardener was conducted on thinner plates (two to four fabric layers), with the researchers pointing out the influence of the composite thickness, impact velocity, and size of the hemispherical striker [52].

Low-velocity impact experiments were carried out on E-glass/epoxy composites of different thicknesses, at sub-zero and elevated temperatures, using a 16 mm diameter hemispherical impactor with impact energies of 50–150 J (keeping v_0_ = 4.44 m/s). The effect of temperature on energy absorption was negligible, but it did have an influence on failure behavior and displacement. The maximum force increased almost linearly with an increase in composite thickness from 5–10 mm. However, it was decreased by 25% when the temperature increased from 20 °C to 100 °C. The laminate thickness had a significant influence (compared to temperature) for the studied range [53]. This research encouraged us to enlarge the range of impact energy to 200 J for the designed composites.

This state-of-the-art for research on the impact behavior of fiber composites underlines the variety of tests conducted (including material architecture, impact energy, and velocity), but also that they are oriented to actual applications. Although many references were analyzed, no research came close to the test campaign designed by the authors in the current study. However, this documentation was useful in disclosing parameters of interest in evaluating the behavior of glass fiber composites.

This paper presents the parametric evaluation of four composites based on the same glass fiber fabric as described in previous works, but having as a matrix two different resins and with and without a middle layer mat. Impact tests conducted with a hemispherical striker of 16 mm revealed differences and similarities in the behavior of these composites.

## 2. Materials, Composite Fabrication, and Test Campaign

A feature of many statistically designed experiments is that they investigate the synergic influence of several variables on the performance of a system. This is achieved by simultaneously changing the values of the variables. This approach is better than the alternative of analyzing each variable individually in a series of separate experiments, because a clearer understanding of the system characteristics and the synergistic way in which they interact can be obtained. Most designed experiments use only 2 or 3 levels, and rarely 4 or 5 levels for their factors to keep the experiment size practicable [54].

Glass fiber fabrics are, by far, the most used in marine systems [55]. The E and S classes are the most commonly available, but at different prices; higher for the S class. The matrices of marine composites are usually made of polyester, vinyl ester, or epoxy resins. In 1973, their percentage in marine composites were ~45%, 20–30%, and 12–15%, respectively [56], because of the relatively high prices for epoxy resins at that time. This trend has been continuing, but the price differences are lower.

Quadriaxial fabrics are integral in manufacturing various ship sizes, from small boats to larger ones. Their multi-directional reinforcement makes them suitable for hull construction, providing structural integrity and resistance to impact and fatigue; decks and superstructures, ensuring stiffness and durability under operational loads; and internal components, such as bulkheads and floors, where strength and weight considerations are critical. Entire hulls can be fabricated from unidirectional reinforcements when an ultra-high-performance laminate is desired [55].

**Figure 1:**
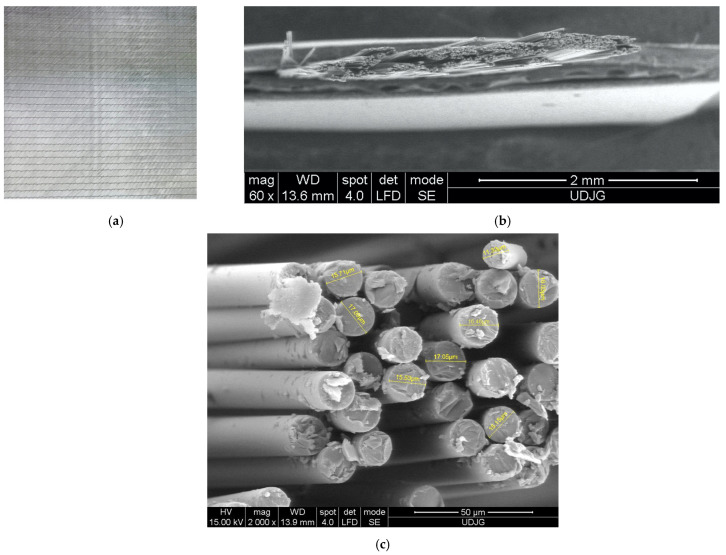
The quadriaxial fabric used in this study: (**a**) general view [57]; (**b**) a single yarn; (**c**) detail with a bunch of glass fibers [58].

The fabric used in this study is a layer of four substrates with an orientation of 0°/+45°/90°/−45° (Figure 1a), which assumes from the outset that the fabric will have a quasi-isotropic behavior. The trade name is 1200 g/m^2^ Quadriaxial Glass Cloth (0°/+45°/90°/−45°) 127, with the code WTVQX1200-1 E-glass, Q1200E10Q, supplied by Castro Composites (Porriño, Pontevedra, España) [57]. Figure 1 also presents a detailed view of the yarn (b) and glass fibers (c), cut with scissors, with the measured diameters being in the range of 11.36–17.05 μm.

In Table 1, the fabric reinforcement construction is detailed. The glass fiber rolls were stored in the laboratory at a relative humidity of 40–70% and a temperature of 18–30 °C, as recommended by the manufacturer.

**Figure 2:**
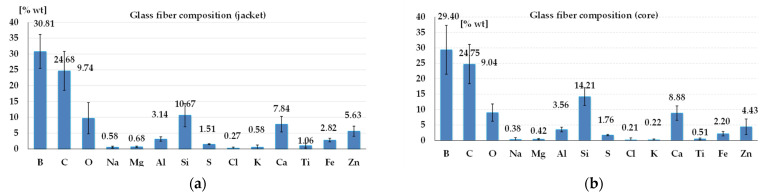
Elemental composition (wt%) of the glass fibers: (**a**) jacket; (**b**) core.

The composition of the glass fibers is given in Figure 2, and one may notice the very small variations in the elemental composition between the glass fiber core (b) and its jacket (a). These graphs contain the average values and standard deviation for 9 measurements for the core and 4 measurements for the jacket. These measurements were also reported in [52,58] as their research used the same quadriaxial fabric.

**Figure 3:**
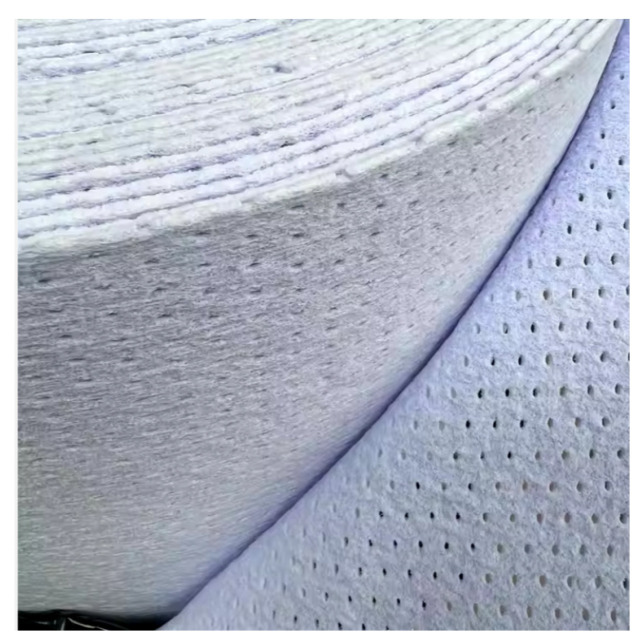
View of Coremat XI 3 mm, selected for the middle layer in the designed composites [60].

After searching through the literature for polyesters compatible with the resins which were going to be used in the fiberglass composite, we selected Coremat^®^ Xi 3 mm (from Lantor, Veenendaal, The Netherlands), supplied by Rompolimer SA (Bucharest, Romania) (Figure 3 and Table 2) [59,60,61,62]. Whether used in the construction of ships, automotive components, leisure equipment, or industrial components, Coremat^®^ Xi 3 offers an acceptable compromise between strength, flexibility, and aesthetics, as a core material and/or a barrier against fiberglass imprinting on the surface of composite end products. It is made of a non-woven polyester with integrated microspheres and is compatible with a wide range of resins including unsaturated polyesters, vinyl esters, phenolics, and epoxy resins [63,64]. One of the main advantages of Coremat^®^ Xi 3 is its excellent impregnation capability with a low amount of resin, which is on average 600 g/m^2^ for 1 mm of thickness.

After searching through the literature for resins for fiberglass composites [64,69] we selected the unsaturated polyester resin Enydyne H 68372 TA (from Polynt Composites, Niepołomice, Poland) [70,71], with Metox-50 W as the accelerator, supplied by Rompolimer Composites [72,73]. The users have to ensure correct curing of the resin for producing durable and strong components. This involves the use of a catalytic system based on PMEC 50% (methyl ethyl ketone peroxide) at 1.5% of the resin mass. The combination of its mechanical strength, thermal stability, and ease of use make it suitable for a wide variety of applications. The physico-chemical and mechanical characteristics of the resin are given in Table 3. Table 4 presents details on co-polimerization process and Table 5 presents characteristics of Metox-50 W The use of Enydyne H 68372 TA resin requires strict safety measures. It contains volatile and flammable monomers such as styrene, which has a flash point of 32 °C, and it is essential that work is carried out in well-ventilated rooms.

The other resin used in this study is a two-component epoxy resin SikaBiresin^®^ CR82 with the hardener CH80-2, supplied by the manufacturer Sika Group (Sika Österreich GmbH, Bludenz, Austria) [77,78], through PolyChem Bucharest, Romania. SikaBiresin^®^ CR82 is suitable for hand brushing and can be used in marine and general industry composites. Material use and processing are recommended at 18–35 °C, and it is certified by Germanischer Lloyd (Hamburg, Germany) (Certificate No. WP 1620020 HH).

An adequate curing cycle for the required mechanical and thermal characteristics depends on various factors, such as the composite thickness, fiber volume, and reactivity of the resin–hardener system. Table 6 shows the SikaBiresin^®^ CR82 resin and the hardener used. A suitable curing cycle may be as follows: heating at a rate of about 0.2 °C/min to ~10 °C below the required glass transition temperature, holding the composite in the mold at this temperature for 2–12 h, followed by cooling at a rate of ~0.5 °C/min.

With the SikaBiresin^®^ CH80-2 and Biresin^®^ CH80-2 hardener (Sika Österreich GmbH, Bludenz, Austria), also supplier by PolyChem Bucharest, Romania, the sample can be removed from the mold at room temperature. The temperature in the composite core can reach 200 °C for a few minutes, after which the composite cools down quickly, reaching room temperature after 5–6 h. Table 7 shows the mechanical and thermal properties of the already formed and heat-treated resin. It can be seen that by using the Biresin^®^CH80-2 hardener, a rather high tensile strength is obtained for an epoxy resin.

**Figure 4:**
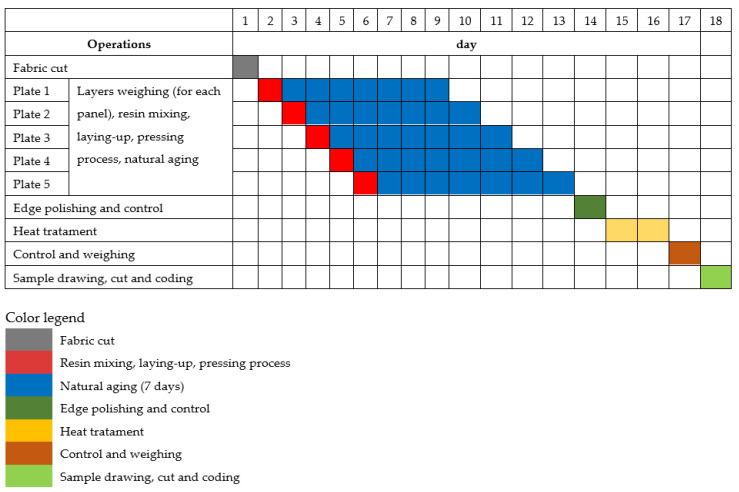
Gantt diagram for fabrication planning of five panels of the same composite.

Hand laminating is a time-consuming but effective method that is still widely used for prototyping and small batch production [85]. Each type of composite (five plates of 300 mm × 300 mm; three plates for impact tests, and two plates for bending tests) could be produced for further characterization, with the steps and duration of manufacturing outlined in the Gantt diagram in Figure 4. At laboratory scale, for producing a set of five plates, the required time was 18 days.

The aim of the laboratory-scale manufacturing process was to produce composite plates that would be suitable for the shipbuilding industry. Before fabricating the composite, the mold was coated with CIREX CP 10, an extraction wax (supplier Airétec, distributor RomPolimer Composites, Bucharest, Romania) [86] used for polyester, gelcoat, and epoxy resins. It is a soft release agent, which evaporates at room temperature and forms a thin monomolecular layer that adheres to the mold walls and has no affinity for the resin or the composite. Each layer of fabric was placed in the press mold and impregnated with resin by manual laying-up. The mat was laid up with resin in the same way. Then, a uniform pressure was applied (0.5–0.7 MPa) for at least 8 h, and the composite plate was demolded and left for natural aging for 7 days at room temperature (20–23 °C). A cure process of holding the plates at 60 °C for 6 h was selected.

**Figure 5:**
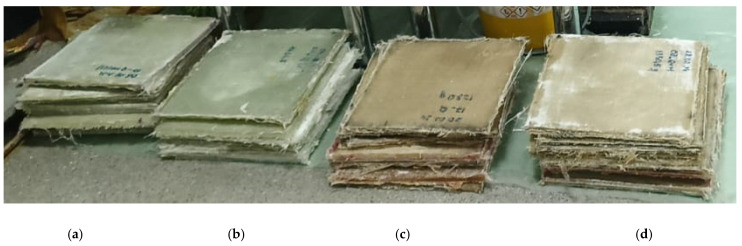
The composite plates after extraction from the press; following extraction, the edges were finished and the plates were weighed. (**a**) QM-c, (**b**) Q-c, (**c**) QM-g, (**d**) Q-g.

Four types of composite materials were obtained (Figure 5). Each plate had dimensions of 300 mm × 300 mm, with similar architectures but different constituents, as follows:

- Q-g: 8 layers of fiberglass fabric, with epoxy resin SikaBiresin^®^ CR82 and hardener CH80-2 as matrix);
- QM-g: 4 layers of fiberglass + 1 layer of Coremat^®^ XI 3 (in the middle) + 4 layers of fiberglass, with epoxy resin SikaBiresin^®^ CR82 and hardener CH80-2;
- Q-c: 8 layers of fiberglass with unsaturated polyester resin Enydyne H 68372 TA, with accelerator Metox-50 W;
- QM-c: 4 layers of fiberglass + 1 Coremat^®^ XI 3 layer + 4 layers of fiberglass with unsaturated polyester resin Enydyne H 68372 TA, with accelerator Metox-50 W.

Tests were carried out on the drop-test machine Instron CEAST 9350 (Norwood, MA, USA) [87] (from INCAS Bucharest, Romania), with a hemispherical steel impactor of 16 mm in diameter and a hardness of 60–62 HRC. Impact tests were carried out for four impact energy values (50 J, 100 J, 150 J, and 200 J), with an impact velocity varying in the range of 4.32–4.42 m/s.

Samples had dimensions of 90 mm × 90 mm, with thicknesses of approx. 8 mm for composites without mat and approx. 11 mm for the composites with Coremat^®^ Xi 3. One might say that the samples are quite small, and that by increasing their size, their behavior could change. A previous discussion on this subject [88] pointed out the influence of scale and size on the mechanical characterization of composites made of hand-laminated unidirectional laminates, as is the case with our designed composites, but the conclusions were based only on tensile and flexural tests. Almost 10 years later, the team [89] carried out impact tests with different hemispherical strikers on samples of different sizes. The results revealed that the impact characteristics scaled well, but only for an elastic domain of loading. For larger samples, failure occurred at a lower load, and displacement and absorbed energy both showed higher levels before fiber failure. This earlier study on scaling the impact behavior of unidirectional glass fiber composites underlined that the first test campaign for new composite recipes should be started on small samples, but should be followed by others with larger sizes (especially surfaces) for producing a prototype with a higher reliability for behavior under impact.

**Figure 6:**
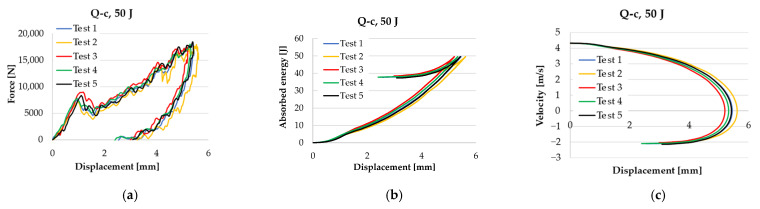
Five tests on samples made of the same composite Q-c: (**a**) force–displacement diagrams, (**b**) absorbed energy–displacement, and (**c**) velocity–displacement.

The tests for each composite type and energy level were repeated five times. If the shape and values of a test differed too much, the test was repeated with new samples until the extremes were eliminated and five tests still remained with close characteristics. As can be seen in Figure 6, the repeatability was of good quality. The first peak force can be generated by the initial delamination of the composite. The wavy shape of the force–displacement curves is typical for composites, and small peaks may indicate damage in yarns, layers, or sublayers.

Drop weight tests are used to evaluate the composite response to a known impact (energy level, velocity, striker shape), especially for automotive and sports equipment industries, but also for composites used in the shipbuilding industry, pipe systems, and aero-space components [90]. The international standards for this test method, ISO 6603-2:2023 [91], describes a test method for both thermoplastic and thermosetting molding and extrusion materials, but also for ”fiber-reinforced thermoset and thermoplastic composites incorporating unidirectional or multi-directional reinforcements such as mats, woven fabrics, woven rovings, chopped strands, combination and hybrid reinforcements, rovings, milled fibers, and sheets made from pre-impregnated materials (prepregs)”. The test parameters for the campaign presented in this study are given in Table 8.

The impact energy is calculated as a potential energy

E_calculated_ = m_impactor_ · g · h_impact_ (1)

where m_impactor_ is the mass of the impactor, with the first value of 5.277 kg being without supplementarily added masses; h_impact_ is the measured drop height (without taking into account the sample height); and g = 9.80665 m/s^2^ is the gravitational acceleration. A percentage difference between the nominal energy and the calculated energy was calculated using the following equation:

ΔE(%) = (E_N_ − E_calculated_) · 100/E_N_ [%] (2)

When the sample is fixed in the test device, the impact energy (as potential energy) is reduced, as the drop height is now h_impact_ − h_sample_. The modification of impact energy due to the sample positioning in the test device can be calculated as:

ΔE(h_sample_) = m_impactor_ · g · h_sample_ (3)

where h_sample_ is the sample height in m, a dimension measured in the impact direction. Table 8 presents the value for ΔE(h_sample_) calculated for the maximum value of the tested samples at each nominal energy level, and the percentage of this value, ΔE%(h_sample_), taking into account the nominal energy. For all tests, the maximum modification of the nominal impact energy was only 1.25%.

The measured parameters were time (sampling time of 0.01 ms), force (with force transducer of 45 KN,, displacement, strain (with data Acquisition system DAS 64 K and a strain gauge of 4 MHz), and velocity, all measuring systems being incorporated in Instron CEAST 9350 drop test machine [92]. The calculated parameter was E_rebound_ (t), meaning the rebound energy at moment t when the impactor was no longer in contact with the sample:

E_rebound_ (t) = 0.5 · m_impactor_ · [v(t)]^2^ (4)

where m_impactor_ is the mass of the impactor (in kg) and v(t) is the impactor velocity (in m/s) at moment t, when the impactor is no longer in contact with the sample.

Several research papers have reported the influence of fiber volume fraction (or fiber mass fraction) on the mechanical properties of glass fiber composites [93,94,95,96] and even on fatigue behavior [97]. The influence of fiber volume fraction was reported by Qiao Y. et al. [98] for a larger range (5–50%), but only for tensile tests with a low test rate (0.2 mm/min). We calculated this feature of the designed composites (Table 8), for each 300 mm × 300 mm composite plate, in order to evaluate the change in fiber volume fraction when adding the middle layer of Coremat^®^ Xi 3. The relationships for calculating the fiber volume fraction, V_f_, are

- for composites without mat

V_composite_ · ρ_composite_ = V_f_ · ρ_f_ + m_resin_ (5)

- for composites with mat

V_composite_ · ρ_composite_ = V_f_ · ρ_f_ + m_mat_ + m_resin_ (6)

with V_composite_ being l^2^ · h_average,_; l being the size of the square plate (considering l = 300 mm after finishing the plate edges); h_average_ being the average height of the plate; and · ρ_f_ being the fiber density (considering · ρ_f_ = 2540 kg/m^3^ for glass fibers). With these in mind, the fiber volume ratio can be calculated as:

V_f/c_ = V_f_/V_composite_ (7)

Using the values in Table 8, the fiber volumes of each tested plate can be calculated, resulting in an average of 0.434 and 0.535 for the composites without mat (Q-c and Q-g, respectively) and 0.329 to 0.338 for the composites with mat (QM-c and QM-g, respectively). The composite QM-c (with mat) has the fiber volume fraction reduced with 24% as compared to composite Q-c (without mat). The composite QM-g (with mat) has the volume fiber fraction reduced with 36.8% as compared to the composite Q-g (without mat). These lower values for the composites with mat explain the more elastic behavior of these composites and the lower absorbed energy values.

The data in Table 9 show the characteristics for the 300 mm × 300 mm plates. From each plate, we could cut 9 samples of 90 mm × 90 mm. Thus, from each set of 3 plates (with the same materials and technology) there were 27 samples in total. For the four tested impact energy values, we needed 4 × 5 samples = 20 samples for each test and material.

## 3. Results

**Figure 7:**
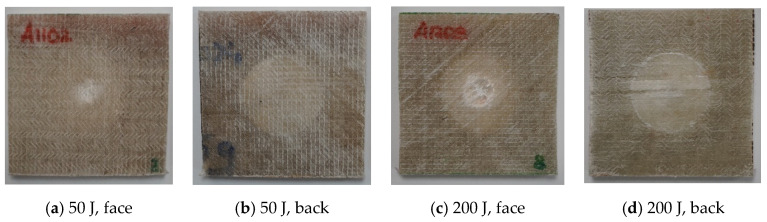
Photos at macrolevel for the composite Q-g, at impact energies of (**a**,**b**) 50 J and (**c**,**d**) 200 J.

**Figure 8:**
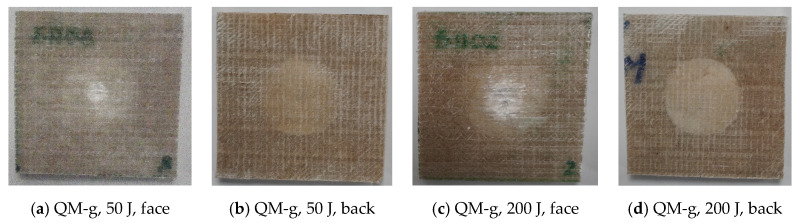
Photos at macro-level for the composite QM-g, at impact energies of (**a**,**b**) 50 J and (**c**,**d**) 200 J.

**Figure 9:**
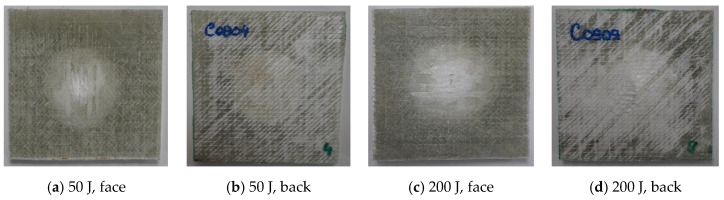
Photos at macrolevel for the composite Q-c, at impact energies of (**a**,**b**) 50 J and (**c**,**d**) 200 J.

**Figure 10:**
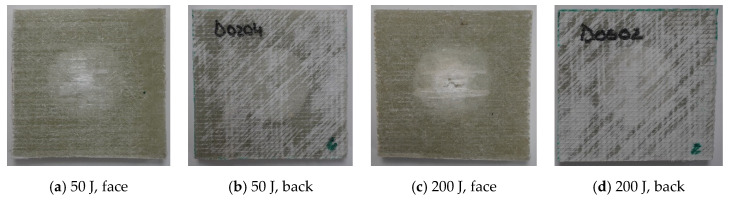
Photos at macrolevel for the composite QM-c, at impact energies of (**a**,**b**) 50 J and (**c**,**d**) 200 J.

Figure 7, Figure 8, Figure 9 and Figure 10 present photos (front and back views) for the extreme values of impact energy, 50 J and 200 J, respectively, for each material. It is obvious that a higher impact energy generates a larger and deeper print on the composite. Close inspection reveals a color change, especially on the back of the sample, which may be a consequence of the delamination process. The sample fixation system was made of metal plates, with a cut of 40 mm in diameter and the sample was fixed under a pressure of 0.3 MPa, that reduces the delamination zone for all tested materials. As can be seen in the figures, the yarns on the first sublayer are fractured and the imprint circle is clearly visible for tests under 200 J. Delamination of the first layers is more visible on the front view of the Enydyne H 68372 TA composite. It is also evident that the damaged area is different for the two resins. The composite with SikaBiresin resin presents fractured yarns, with the line of these fractured yarns being almost perpendicular to the yarns’ direction. The composites made with Enydyne H 68372 TA has a “star” fracture for the tests with 200 J. The composites Q-g and QM-g seem to have less intense damage for the 200 J tests. For tests at 50 J, the damaged surfaces are quite similar for all tested composites. On the backs of the composites, there is no visible damage of the last layer, only a light color presuming delamination in the free zone, having approximately the diameter of the hole in the fixing plates. For tests at 200 J, the composites without mat present a visible central band of delamination in the last layer. Delamination is a critical failure in composites and depends on many factors including composite constituents and their architecture, the fabrication process, and the size and shape of the actual composite body [96,97,98,99].

**Figure 11:**
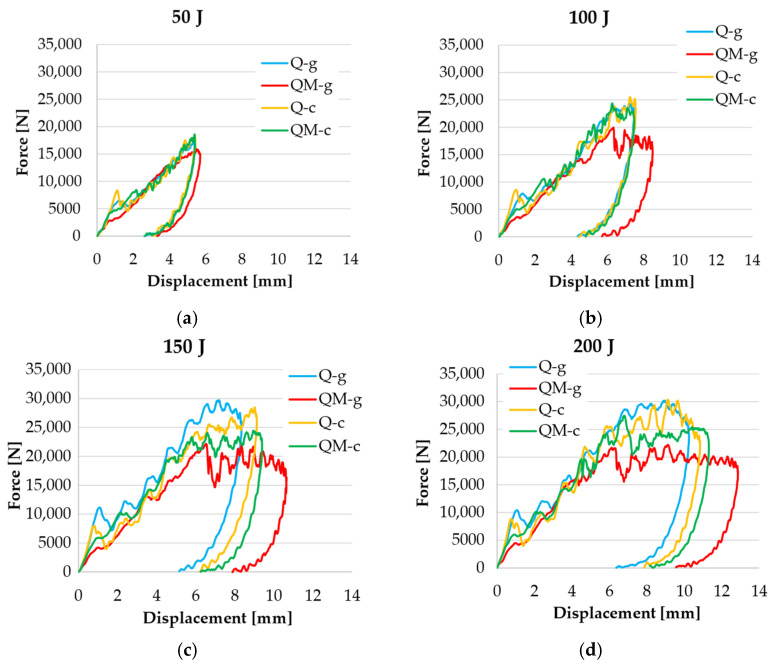
Typical force–displacement curves for each impact energy level: (**a**) 50 J; (**b**) 100 J; (**c**) 150 J; (**d**) 200 J.

By superimposing these curves for each designed composite, one may differentiate the behavior of the four tested composites at each impact energy level. By examining the overlaid curves (Figure 11), trends and patterns were noticed, revealing the material response to increasing impact energy. The force–displacement curve is an important characteristic for assessing the impact damage resistance of composites. The curve provides information about the energy absorption capacity of the laminate (the area under the force evolution), the maximum force that the sample can withstand, and the deformation behavior of the laminate during the impact process.

The force–displacement response is characterized by three distinct stages. In the first stage, the force increases with small oscillations that could be the result of successively damaging layers or sublayers of quadriaxial fabric. The second stage is a plateau (not visible for the 50 J tests, for any of the four composites), visibly larger for the composite QM-g. This starts, finishes, or contains the value of F_max_. During the third stage, the force decreases rapidly, reaching the null value for d_f_ (final displacement before impactor detachment from the sample), corresponding to the first value of F = 0 (after F_max_). Ge X. et al. [100] tested composites made of carbon fibers and vinyl ester resin, with a similar range of impact (16 J–283 J), with similar thicknesses for 30 layers of woven fabric, and obtained force–displacement curves with a “saw teeth” shape. These comparisons can help practitioners in selecting the appropriate composite based on impact behavior, but also considering the price or relative density of the composites. Assae Z. et al. [101] obtained similar shapes for hybrid composites (bi-axial glass fiber fabrics and magnesium alloy sheets), meaning that the fiber composite layers fail successively.

**Figure 12:**
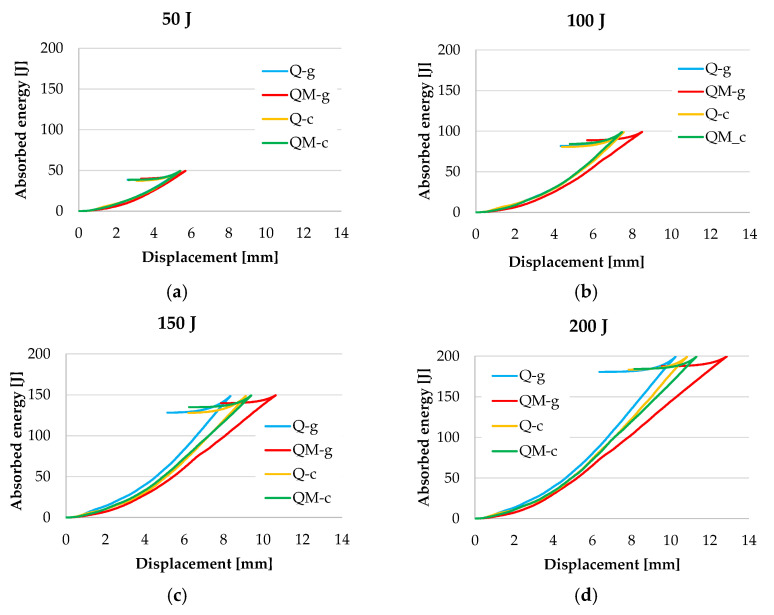
Typical absorbed energy–displacement curves for each impact energy level: (**a**) 50 J; (**b**) 100 J; (**c**) 150 J; (**d**) 200 J.

Figure 12 presents the graphics of absorbed energy–displacement curves in order to highlight the comparison of this parameter among the four composites. For the lowest tested impact energy of 50 J, all the curves almost overlap, meaning the response of the composites is almost identical. These curves could also be analyzed as a three-stage process. In the first stage, the absorbed energy values were low due to shallow deformation in the sample thickness direction under the transverse impact load and the elastic displacement of the plate. At the second stage, the energy–displacement curves show an increase in slope, representing an increase in sample deformation, with possible internal damage; the energy absorption is higher for smaller displacements, meaning a more intense energy transfer in the in-plane direction. At a certain moment, the entire impact energy is transferred to the plate, but the elastic component of this energy tries to bound the impactor until the contact with the plate vanishes (i.e., F_f_ = 0). The impactor regains a part of its impact energy, which will have a constant evolution immediately after detaching from the plate. Its evolution is stopped by the protection system of the test machine.

**Figure 13:**
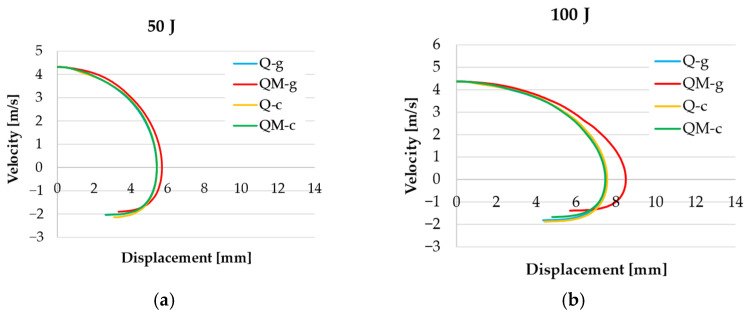

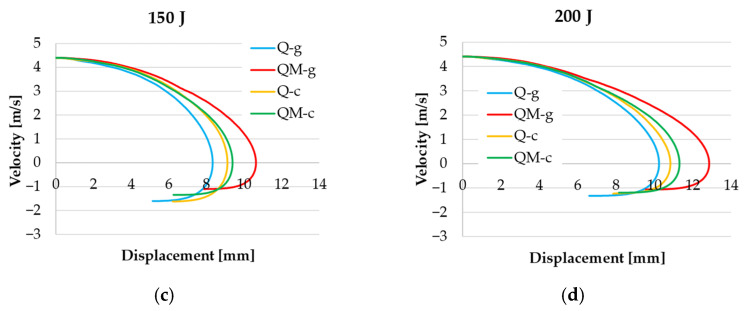
Typical velocity–displacement curves for the tested composites: (**a**) 50 J; (**b**) 100 J; (**c**) 150 J; (**d**) 200 J.

Figure 13 presents velocity–displacement curves. For 50 J, there are no significant differences, meaning that the impact response is hard to be differentiated among the four composites. For 100 J, only the composite QM-g has a larger displacement for v = 0.

**Figure 14:**
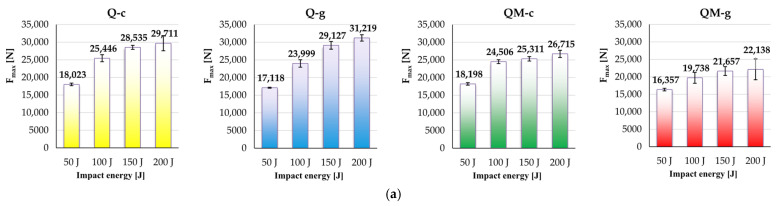

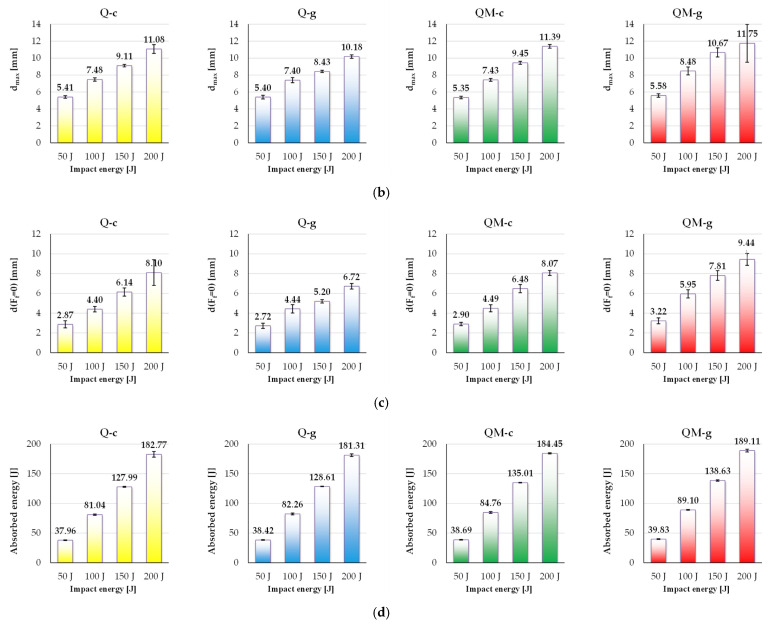
Measured impact parameters: (**a**) maximum force (F_max_); (**b**) maximum displacement (d_max_); (**c**) displacement at impact end, noted as d(F_f_ = 0); (**d**) absorbed energy; color legend: yellow—composite Q-c, blue—composite Q-g, green—composite QM-c, and red—composite QM-g.

The following plots in Figure 14 analyze the dependence of several parameters characterizing the impact on the impact energy and composite recipe, taking into account the average values for the five identical input tests:

(a) F_max_ increases with impact energy, but this increase depends also on composite type: both composites without Coremat^®^ Xi 3 (Q-c and Q-g) have higher values for F_max_ as compared to the composites with mat (QM-c and QM-g); the dependence on impact energy is more pronounced for composites without mat; for composites with mat, the slope of this dependence is lower.
(b) Maximum displacement (d_max_) has average values very close for the 50 J test for all composites, but when increasing the impact energy, this parameter increases in the same manner for every impact energy level: d_max (Q-g)_ < d_max (Q-c)_ < d_max (QM-c)_ < d_max (QM-g)_.
(c) Displacement at the end of the impact, when F_f_ = 0 after reaching the maximum value, noted as d(F_f_ = 0), could be ordered as d(F_f_ = 0)_(Q-g)_ < d(F_f_ = 0)_(Q-c)_ < d(F_f_ = 0)_(QM-c)_ < d(F_f_ = 0) _(QM-g)_ for all impact energy values.
(d) For the energy absorption, the behavior of the composites is clearly divided into two groups: the two composites with the epoxy matrix have lower values, and those including the mat in the middle of the composites exhibited the highest values; however, differences are too small to make this parameter a well-defined influence factor.

Table 10 presents comparisons of the experimental results, as found in open sources. From the analyzed documentation, one may conclude that tests on thicker composites and high impact energies are rare, but are still of interest to the designer because the thickness of the obtained composites could make them an option for marine industries.

**Figure 15:**
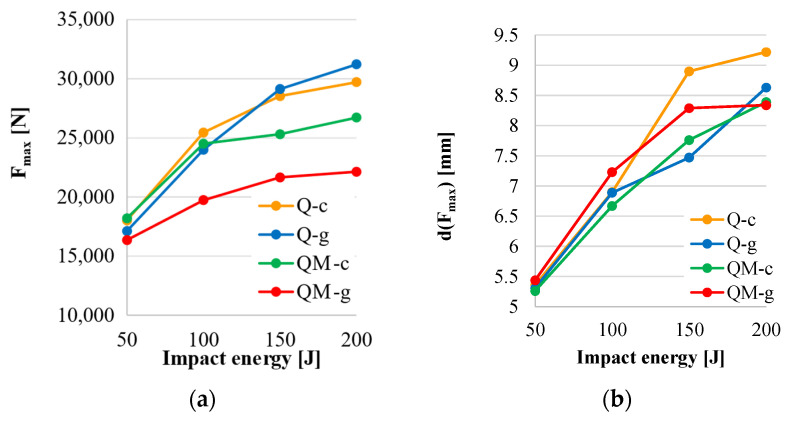

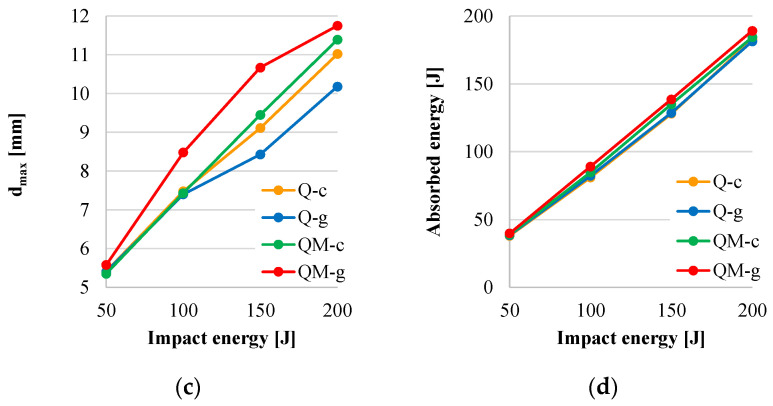
The dependence of impact parameters on impact energy: (**a**) maximum force, F_max_; (**b**) displacement at F_max_; (**c**) maximum displacement, d_max_; (**d**) absorbed energy.

A synthesis of the experimental data is discussed based on the graphs in Figure 15.

The highest value for F_max_ was obtained at 150 J and 200 J for the composite Q-g; all composites increased in F_max_ with impact energy, but with different curve shapes; the lowest values were obtained for QM-g (Figure 15a).

The displacement at F_max_, d(F_max_) increased with the same tendency until 100 J, but, for the following values of the impact energy, the evolution was not obviously ordered (Figure 15b). The composite QM-g had the largest maximum displacement values (Figure 15d); the composites with the unsaturated polyester resin had a similar evolution of this parameter, but the composite Q-g was the most rigid (with the lowest values for d_max_) for 150 J and 200 J.

Composite QM-g had the highest absorbed energy values, but we consider the plots to be too close to have a distinct recommendation for this composite (Figure 15d).

Based on this analysis, the authors concluded that for selecting one of the four designed composites, the user has to analyze other parameters, including costs with materials and manufacturing, and durability in particular environments for ships (salt or freshwater, cold or warm environment, etc.).

## 4. Conclusions

This study presents a comparison among the impact results for four composites, designed by the authors and fabricated at laboratory scale, based on the same quadriaxial glass fiber fabrics as previously produced composites, but with two different resins and introducing a mat layer in the middle of each composite. The study analyzed several parameters characterizing the experimental work. Although the tested impact energy range was quite large (50–200 J), only partial penetrations for all tested composites were obtained.

When comparing force–displacement, absorbed energy–displacement, and velocity–displacement curves, for the lowest tested energy (50 J), all composites had a similar behavior, but for the higher impact energy values (100–200 J) there was a visible difference between the two composites without mat and the two composites with mat.

At low-velocity impact, the composites without mat could be ranked: Q-g had better properties than Q-c, but the results were close.

The composites with mat (QM-g, QM-c) had no clear differentiation in their properties. They absorbed slightly more energy compared to those without the mat (Q-g, Q-c). The increase in energy absorption was approximately 0.91–4.3% (at an impact energy of 200 J), a marginal improvement. The mat layer contributed to enhanced energy dissipation, demonstrating slightly more elastic behavior during impact. At lower impact energies (50 J), the differences in energy absorption were not visible. However, at higher energies (100–200 J), the influence of the mat layer became visible.

Composites QM-g and QM-c exhibited significantly lower values for maximum force during impact as compared to those without the mat (Q-g, Q-c). This can be attributed to the fact that the mat layer disperses stress more effectively, reducing the peak force during the impact. This reduction is a critical advantage, as it minimizes the probability of localized damage initiation or propagation. The effect of the mat layer on maximum force was consistent across both resin types, indicating its dominant role in modifying impact behavior.

Composites with epoxy resin (Q-g, QM-g) outperformed those with unsaturated polyester resin (Q-c, QM-c) for all characteristics, due to the superior mechanical characteristics of the resin. However, the relative improvements provided by the mat layer were observed regardless of resin type, but specifically for higher impact energies (100–200 J).

At lower impact energies (50 J), no visible differences in performance were observed, likely due to the smaller forces involved being insufficient to highlight the contributions of the mat or resin’s properties. At higher impact energies (100–200 J), the distinctions in energy absorption and elasticity became apparent.

Although there were variations in energy absorption due to the mat, its practical significance was limited, suggesting that the choice to include a mat layer might depend on specific application requirements rather than clear performance superiority in low-velocity impacts.

It can be concluded that the lower maximum force in composites with the mat layer makes them more suitable for applications requiring resilience to high-energy or repetitive impacts.

The results suggest that the inclusion of a glass fiber mat layer in the middle of the tested composites provides modest benefits in terms of energy absorption in low-velocity impacts and a significant reduction in maximum force. These properties could be advantageous in specific applications, especially when combined with a high-performance resin. However, the practical significance of the observed differences should be evaluated based on specific use-case requirements.

## Figures and Tables

**Table 1:** Characteristics of the fabric architecture [57].

Sublayer	Layer Orientation	Fiber Type [Tex]	Area Density [g/m^2^]
1.	0 °C	600	283
2.	45 °C	300 + 600	300
3.	90 °C	600	307
4.	−45 °C	300 + 600	300
	Auxilliary yarn	76 DTex	10
Total: 1200 g/m^2^ (±%3)			

**Table 2:** Characteristics of Coremat^®^ Xi 3 [59].

Property	Value	Standard/Test
Thickness, mm	3.0	-
Area density (dry), g/m^2^	80	-
Flexural strength, MPa	11	ASTM D790 [65]
Flexural modulus MPa	1100	ASTM D790 [65]
Tensile limit between layers, MPa	4	ASTM C297 [66]
Compression limit (at 10% strain), MPa	10	ISO 844 [67]
Shear strength, MPa	5	ASTM C273-61 [68]
Shear modulus, MPa	35	ASTM C273-61 [68]

**Table 3:** Characteristics of resin Enydyne H 68372 TA [70].

Characteristic	Value
Density (at 20 °C)	1.10 g/cm^3^
Resin content	57–63%
Brookfield viscosity RVT (at 23 °C)	4–6 dPa.s
Working method	R 151
Working temperature	23 °C
Catalytic system	1.5% PMEC, 50%
Tested mass resin	100 g
Maximum temperature at peak reaction	<135 °C
Tensile strength (ISO 527) [74]	45 MPa
Strain at break	1.5%
Flexural strength (ISO 178 [75])	65 MPa
Flexural modulus	3300 MPa
Heat deflection temperature (HDT ISO 75-2 A [76])	70 °C

**Table 4:** Co-polymerization process: resin (100 g) and hardener (2 g) [72].

Hardener	Gel Time [min]	Peak Temperature [°C]	Time to Peak Temperature [min]
Metox-50 W	27–28	145.0–149.4	46–48

**Table 5:** Characteristics for Metox-50 W [73].

Characteristic	Value
Active oxygen, %	8.5–8.9
Density, g/cm^3^	1.170–1.176
Peroxides, %	30–35
Self-accelerating decomposition temperature (SADT), °C	60
Gel time, min	28–29
Storage temperature, °C	0–25
Time till maximum temperature, min	40–42
Water content, approx., %	approx. 2.0

**Table 6:** Characteristics of the epoxy resin SikaBiresin^®^ CR82 and hardener Biresin^®^ CH80-2 [77].

Characteristic	Measurement Unit	Resin SikaBiresin^®^ CR82	Hardener Biresin^®^ CH80-2
Mixing ratio	massic	100	27
Viscosity, at 25 °C	mPa.s	~1600	
Density, 25 °C	g/mL	1.11	0.99
Potlife, 100 g/RT, approx. min			80
Viscosity of the mix, mPa.s 25 °C (approx.)		600	

**Table 7:** Mechanical properties of the epoxy resin SikaBiresin CH80-2 [77].

Property	Method	Value
Tensile strength, MPa	ISO 527 [74]	90
Young modulus, MPa	ISO 527 [74]	3000
Strain at break, %	ISO 527 [74]	5.6
Flexural strength, MPa	ISO 178 [75]	130
Flexural modulus, MPa	ISO 178 [75]	3200
Compression strength, MPa	ISO 604 [79]	105
Density, g/cm^3^	ISO 1183 [80]	1.14
Shore hardness, D	ISO 868 [81]	85
Impact resistance, kJ/m^2^	ISO 179-2 [82]	66
Glass transition temperature by DSC, °C	ISO 11357-1 [83]	89
Heat deflection temperature, °C	ISO 75-1 [84]	83

**Table 8:** Values of test parameters.

E_N_ [J]	h_impact_ [m]	m_impactor_ [kg]	E_calculated_ [J]	E_N_ − E_calculated_ [J]	ΔE(%) [%]	h_sample max_ [mm]	ΔE(h_sample_) [J]	ΔE%(h_sample_) [%]
50	0.966	5.277	49.990	0.010	0.019	12.049	0.624	1.25
100	0.992	10.277	99.977	0.023	0.023	11.901	1.199	1.20
150	1.001	15.277	149.966	0.034	0.022	11.234	1.683	1.12
200	1.006	20.277	200.043	−0.043	−0.021	11.563	2.299	1.15

**Table 9:** Characteristics of the fabricated plates at laboratory scale.

Plate	Fabric Mass, m_f_ (g)	Composite Plate Mass (g)	Resin Mass * (g)	Mass Fiber Ratio (m_f_/m_composite_)	Surface Density (m_composite_/A_composite_) (kg/m^2^)	V_f/_V_composite_	h_1_ (mm)	h_2_ (mm)	h_3_ (mm)	h_4_ (mm)	h_average_ (mm)
0	1	2	3	4	5	6	7	8	9	10	11
Q-c-08	839.5	1417.5	578	0.592	15.75	0.409	8.75	9.1	8.9	9.2	8.988
Q-c-09	861	1347	486	0.639	14.967	0.467	8.2	7.95	7.8	8.3	8.063
Q-c-12	834.5	1372.5	538	0.608	15.25	0.426	8.65	8.4	8.7	8.55	8.575
Average Q-c	845	1379	534	0.613	15.322	0.434	8.5	8.5	8.5	8.7	8.542
Q-g-11	855.00	1254.5	399.5	0.682	13.939	0.447	8.4	8.45	8.35	8.25	8.363
Q-g-12	844.50	1351.5	507	0.625	15.017	0.428	8.65	8.7	8.6	8.55	8.625
Q-g-14	846.50	1300	453.5	0.651	14.444	0.429	8.6	8.55	8.65	8.75	8.638
Average Q-g	848.67	1302.00	453.33	0.65	14.47	0.435	8.55	8.57	8.53	8.52	8.54
QM-c-02	843.00	1555	704.00	0.547	17.278	0.321	11.65	11.2	11.4	11.7	11.488
QM-c-05	848.00	1534	678.00	0.558	17.044	0.336	10.9	11.1	10.75	11.45	11.05
QM-c-06	844.00	1543	691.00	0.552	17.144	0.329	10.9	11.45	11.25	11.25	11.213
Average QM-c	845.00	1544.00	691.00	0.55	17.16	0.329	11.15	11.25	11.13	11.47	11.25
QM-g-02	831.00	1361	521.50	0.617	15.122	0.331	11.25	11.55	10.35	10.8	10.988
QM-g-05	858.00	1362.5	497.50	0.635	15.139	0.342	11.2	10.7	11.25	10.75	10.975
QM-g-06	860.00	1353	485.00	0.642	15.033	0.340	11.2	11.1	10.9	11.1	11.075
Average QM-g	849.67	1358.83	501.33	0.631	15.10	0.338	11.22	11.12	10.83	10.88	11.01

* m_resin_ = m_composite_ − m_fabric_ (i.e., Column 2–Column 3) for composites Q-c and Q-g and m_resin_ = m_composite_-m_fabric_ − m_mat_; m_mat_ ~8 g for each layer of 300 mm × 300 mm; A_composite_~0.09 m^2^.

**Table 10:** Experimental works from the open literature for impact tests on composites.

Reference	Composite	Sample Dimensions	Test Machine, Steel Impactor Size, Shape, Mass, Repeated Tests	Impact Energy, Velocity, Temperature
2019 [48]	Glass fibers with different matrix: epoxy, polyester, Elium-acrylic, Elium-acrylic + 10 wt% of Nanostreh tri-block copolymers) and (Elium-acrylic + 10 wt% ABS	100 mm × 150 mm, 2 mm thickness	Hemispherical striker, diameter 16 mm, and mass of 0.1255 kg, 3 tests	10–60 J
2019 [53]	Epoxy resin, hardener (LY556, HY5200), E-glass plain woven roving [0/90], 0.25 mm thickness, 360 g/m^2^	5 mm, 7 mm, and 10 mm thickness,	Hemispherical steel impactor of 16 mm diameter	50–150 J, 4.34–7.51 m/s −20 °C, 25 °C, 100 °C
2024 [51]	Unidirectional S2-glass fibre/FM94-epoxy resin prepreg	4.2 mm thickness, 70 mm square	Impactor with a 20 mm spherical diameter	75 J, 150 J, 225 J 25–100 °C,−25–55 °C
2024 [49]	8-Harness Satin S2-Glass Fabrics (302 g/m^2^, 0.24 mm)–elastomer polyurethane resin	Thickness 2.48–6.39 mm	Hemispherical impactor 16 mm, one test!	48.8 ± 0.4 J, 86.2 ± 0.4 J, 115.3 ± 0.9 J
2020 [50]	Carbon fibers, glass fibers, hybrid com-posites, resin: epoxy, phenolic, polyesther	3,3 mm, 4.9 mm, 7.4 mm	Instron Dynatup 9250 G, 25.4 mm hemispherical impactor	20 J 2.06 m/s
2023 [52]	Quadriaxial glass fiber fabric 2, 4 and 6 layers of fabrics Epoxy resin	60 mm × 60 mm 1.65–5.35 mm, 3 times	10 and 20 mm hemispherical impactors	v_0_ = 2 m/s, 3 m/s, and 4 m/s 11 J, 16 J, 45 J
2017 [101]	Hybrid composite: Magnesium metal sheets, 3D fiberglass fabric, epoxy resin, and polyurethane foam	No information on thickness	Modified Charpy striker	3 J, 11 J, 25 J, 45 J, 70 J, 100 J
2022 [100]	Woven carbon (T300-3 K) fabrics twilled 2 × 2, 387 g/m^2^. 60 plies of fabrics, [(0/90)30]_S_, 0.22 mm. Vinyl ester 430 LV resin	~13 mm 150 mm × 100 mm	Instron 9350, hemispheric impactor of 16 mm, total weight of impactor 5.5 kg	From 16–283 J 3 times each test
2022 [20]	Filament-wound composite, sequence [90°_2_/±28°]_3_, T700 carbon fibers and epoxy resin	150 mm × 100 mm 2.1 mm	Instron Dynatup 9250 HV, hemispherical impactor of 12.7 mm, mass 6.5 kg.	5 J, 10 J, 15 J, 20 J 1.24–3.28 m/s
2024 [19]	Yacht bow floor, composites with chop strand and different carbon fibers fabric		Impactor mass of 5.7 kg and diameter of 20 mm	5 J, 10 J, 15 J, 20 J, 25 J, and 30 J

## Data Availability

Data are contained within the article.
